# Origin of phagotrophic eukaryotes as social cheaters in microbial biofilms

**DOI:** 10.1186/1745-6150-2-3

**Published:** 2007-01-19

**Authors:** Gáspár Jékely

**Affiliations:** 1 European Molecular Biology Laboratory, Meyerhofstrasse 1. 69117 Heidelberg, Germany; 2 Collegium Budapest Institute for Advanced Study, Szentháromság utca 2. 1024 Budapest, Hungary

## Abstract

**Background:**

The origin of eukaryotic cells was one of the most dramatic evolutionary transitions in the history of life. It is generally assumed that eukaryotes evolved later then prokaryotes by the transformation or fusion of prokaryotic lineages. However, as yet there is no consensus regarding the nature of the prokaryotic group(s) ancestral to eukaryotes. Regardless of this, a hardly debatable fundamental novel characteristic of the last eukaryotic common ancestor was the ability to exploit prokaryotic biomass by the ingestion of entire cells, i.e. phagocytosis. The recent advances in our understanding of the social life of prokaryotes may help to explain the origin of this form of total exploitation.

**Presentation of the hypothesis:**

Here I propose that eukaryotic cells originated in a social environment, a differentiated microbial mat or biofilm that was maintained by the cooperative action of its members. Cooperation was costly (e.g. the production of developmental signals or an extracellular matrix) but yielded benefits that increased the overall fitness of the social group. I propose that eukaryotes originated as selfish cheaters that enjoyed the benefits of social aggregation but did not contribute to it themselves. The cheaters later evolved into predators that lysed other cells and eventually became professional phagotrophs. During several cycles of social aggregation and dispersal the number of cheaters was contained by a chicken game situation, i.e. reproductive success of cheaters was high when they were in low abundance but was reduced when they were over-represented. Radical changes in cell structure, including the loss of the rigid prokaryotic cell wall and the development of endomembranes, allowed the protoeukaryotes to avoid cheater control and to exploit nutrients more efficiently. Cellular changes were buffered by both the social benefits and the protective physico-chemical milieu of the interior of biofilms. Symbiosis with the mitochondial ancestor evolved after phagotrophy as alphaproteobacterial prey developed post-ingestion defence mechanisms to circumvent digestion in the food vacuole. Mitochondrial symbiosis triggered the origin of the nucleus. Cilia evolved last and allowed eukaryotes to predate also on planktonic prey. I will discuss how this scenario may possibly fit into the contrasting phylogenetic frameworks that have been proposed.

**Testing the hypothesis:**

Some aspects of the hypothesis can be tested experimentally by studying the level of exploitation cheaters can reach in social microbes. It would be interesting to test whether absorption of nutrients from lysed fellow colony members can happen and if cheaters can evolve into predators that actively digest neighbouring cells.

**Implications of the hypothesis:**

The hypothesis highlights the importance of social exploitation in cell evolution and how a social environment can buffer drastic cellular transformations that would be lethal for planktonic forms.

**Reviewers:**

This article was reviewed by Eugene V Koonin, Purificación López-García, and Igor Zhulin.

## Open peer review

This article was reviewed by by Eugene V Koonin, Purificación López-García, and Igor Zhulin.

For the full reviews, please go to the Reviewers' comments section.

## Background

The origin of eukaryotes from prokaryotic ancestors involved profound changes in cellular architecture [1]. The exact order and causation of these changes are still intensely debated [2-6], but there is an emerging consensus regarding the key cellular features already present in the last eukaryotic common ancestor. These include, among others, the presence of mitochondria, a dynamic endomembrane system comprising endosomes, lysosomes, phagosomes, autophagosomes, nuclear compartmentalisation, an endoplasmic reticulum, a Golgi-complex, actin-based lamellipodia, and a centriole-based cilium [7-9].

Although several models have been proposed on the origin of eukaryotes, here I distinguish two major model types that differ in one important aspect regarding the timing of the acquisition of key eukaryotic features. In one model type the primary event in eukaryogenesis is a symbiosis, a merger of two distinct prokaryotic lineages [2,3,10-12]. This symbiotic event, sometimes imagined starting off as a metabolic association [2,3,11], is then thought to have triggered all subsequent cellular changes, including the origin of endomembranes. In the alternative model the development of an endomembrane system, and most importantly of phagotrophy, precedes the symbiotic acquisition of a protomitochondrium [4,5,13].

The recognition that all extant amitochondriate protists once harboured mitochondria [14-18] seemed to tip the balance in favour of symbiosis-first models [2,19]. However, the presence of mitochondria in the last eukaryotic common ancestor (cenancestor) does not necessarily mean that mitochondria came before phagotrophy since phagotrophy was also present in the cenancestor [8]. Phagotrophy-first models are therefore as valid as ever [20,21].

The ancestry of phagotrophy is evidenced by its broad phyletic distribution among eukaryotes [22]. Among Unikonts, representing one major branch of the eukaryotic tree [8], Metazoa, Amoebozoa [23], Choanoflagellates [24], and several other protist groups are phagotrophic [25]. Fungi lost the ability of phagocytosis early in their evolution [26]. The closest known relative to fungi, the amoeboid protist *Nuclearia*, is a phagotroph [25]. Basal fungi can also have amoeboid phases such as the zoospores of some Chytridiomycota [26]. The pathogenic basal fungus *Rozella allomycis *can even phagocytose organelles of its host [27]. Among Bikonts (Plantae, Alveolata, Rhizaria, Excavata, Chromista) [8,28-31], representing the other branch of the eukaryotic tree, phagocytosis is also widespread [22]. With the exception of Plantae all major Bikont groups contain phagotrophic taxa [4,30-33].

If the eukaryotic tree is rooted between Unikonts and Bikonts [8,28], the eukaryotic cenancestor was clearly phagotrophic. This remains true even if the tree is rooted on Diplomonads (e.g. *Enteromonas*, *Giardia*) or Parabasalids (e.g. *Trichomonas*), formerly believed to be early branching, because these taxa are also phagotrophic [34-36]. Based on the presence of a T3/T7-like polymerase in all mitochondrial genomes except Jakobids it has been suggests that these protists may be early branching [37,38]. Jakobids are also phagotrophs [39] so a rooting between them and the rest of eukaryotes would still mean that the cenancestor was phagotrophic.

The ancestral presence of phagotrophy in eukaryotes means that all models of eukaryogenesis have to account for its origins. To date none of the symbiotic scenarios is sufficiently developed to explain why a prior endosymbiosis triggered the development of phagotrophy. If the order of origins is reversed, the problem disappears: phagotrophy can easily account for the acquisition of symbionts (the phagotrophic origin of plastids is generally accepted). Phagotrophy-first models have therefore primarily to account for the origin of an endomembrane system and phagotrophy from a non-phagotrophic prokaryotic ancestor.

Here I present a novel scenario on the origin of phagotrophy and other eukaryotic features that emphasizes the social context of the prokaryote-eukaryote transition. I propose that eukaryotes originated in a multicellular bacterial mat or biofilm through social conflict and a continuing evolutionary arms race. I will show that a social scenario can help to explain both how phagotrophic exploitation originated and how the drastic cellular transitions (e.g. the loss of a rigid cell wall) could have occurred without a severe reduction in fitness of the transitional forms.

It has become increasingly recognised in recent years that a wide variety of prokaryotes lives in biofilms, highly structured multicellular, often multispecies communities. [40-42]. Biofilms are composed of microcolonies encased in an extracellular polymer matrix [43] that form on surfaces during a highly ordered developmental sequence [40]. The formation of biofilms starts with the attachment of cells to the surface and continues with the secretion of an extracellular matrix. Biofilms eventually mature into a stratified structure with marked differences in cellular morphologies and gene expression patterns between cells in the outside and the inside [44,45]. During biofilm maturation the extracellular matrix forms a network of channels facilitating nutrient and water exchange in the interior of the biofilm [40]. From mature biofilms individual cells, spores or cell clusters shed off and disperse to re-initiate the developmental cycle at colonisable surfaces [46-48].

The formation of biofilms is a social strategy that involves a costly contribution from the cooperating individuals and confers a fitness-benefit to the group [49] (but see also [50]). The costs can include the production of a developmental signal, an extracellular matrix, digestive enzymes or altruistic self-sacrifice. The benefits include, among others, a decreased risk of predation, an increased resistance to antibiotics or toxic environmental conditions and more effective resource exploitation [51-54]. In social microbes dispersal is restricted to a fraction of the group and depends on the altruism of non-dispersing colony members. Altruistic members can for example form a fruiting body stalk or supply nutrients to dispersing cells by autolysis. Lysis can be induced either cell autonomously as part of biofilm maturation [47,55] or by the dispersing cells that secrete a killer toxin [56].

As in all social systems the benefits of cooperation can be undermined by selfish 'cheaters' that use, but do not contribute to a collectively produced fitness-enhancing resource. As a result, cheaters can grow faster and can be overrepresented during dispersal. The emergence of cheaters from within a cooperating social group has been described in several cases, including prokaryotic and eukaryotic microbes and viruses [49,57-60]. The analysis of frequency-dependent cheater fitness by evolutionary game theory predicts that some cheater genotypes should persist over long evolutionary time [61]. There is also theoretical [62] and experimental [63] evidence that cheater genotypes can coexist with their social kin over several cycles of competition.

**Figure 1:**
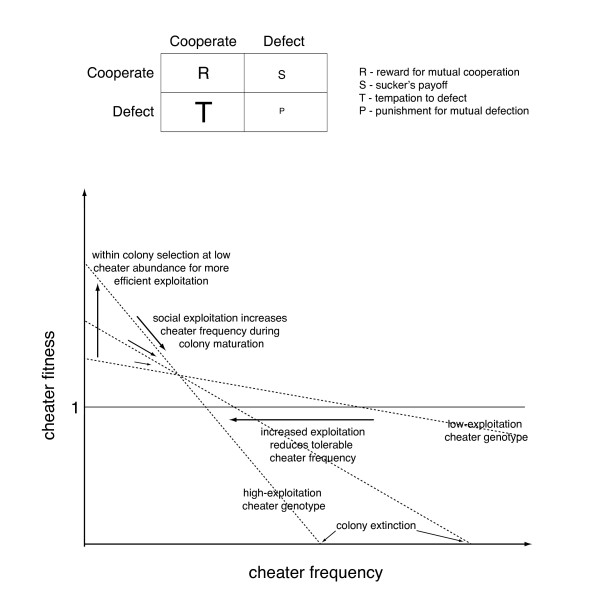
**Proposed fitness relationships during increasing social exploitation by protoeukaryotes**. The fitness matrix of the chicken game (above) shows the relative magnitude of the fitness gain for players with different strategies (defectors or cooperators) depending on the strategy of other players. In the case of social microbes, players can be conceived as subpopulations within a group of cells displaying distinct genotypes and social strategies. The dependence of cheater fitness on cheater frequency (below), corresponding to the fitness matrix of the chicken game, illustrates the selective scenario for the evolution of more potent cheaters.

A game theoretical approach is very useful to analyse the social interactions in microbes [61]. In game theory, interacting players with distinct strategies compete for a fitness enhancing benefit [1,64]. The gain of each player depends on the strategy of other players. In the case of social microbes, instead of considering two players, one can conceive a large group of cells within which subpopulations have distinct genotypes and social strategies [61]. In a social microbial framework, cells can either cooperate (form a fruiting body stalk etc.) or defect and become potential cheaters. The benefit of cooperation vs. defection depends on the frequency of the players exhibiting each strategy and on the extent of defection. This can range from non-contribution to direct exploitation (e.g. predation). The benefit of each strategy can be represented in a fitness matrix (Fig. 1). Mutual cooperators receive a fitness benefit, R, the 'reward for cooperation'. A small group of defectors within a large group of cooperators receives T, the 'temptation to defect'. If defectors are at high abundance, they receive P, the 'punishment for mutual defection'. Exploited cooperators receive S, the 'suckers payoff'. If the fitness enhancing benefit depends on the cooperative action of a social group, the matrix of frequency dependent payoffs is best represented by the 'chicken game' [61]. In the chicken game the benefit of defectors is higher then that of cooperators when they are at low abundance but falls below cooperator benefit when defectors reach a critical abundance (T>R>S>P) [61].

The 'prisoner's dilemma', another matrix of fitness relationships, seems to be less suited to describe the fitness relationships for social microbes. In the prisoner's dilemma, cheating always has a fitness advantage over cooperation (T>R>P>S). This fitness matrix corresponds to cases where an independent resource is being exploited, such as a growth medium by bacteria [59] or a cell's cytoplasm by viruses [60].

The game theoretical framework describes the fitness gain of each genotype during one repetition of the game. During several rounds of social aggregation and dispersal, the game is always repeated and the long-term evolutionary outcome will depend on the fitness matrix. The fitness relationships in the chicken game can maintain genetic polymorphism because both types have a selective advantage when rare [63]. Besides the fitness matrix, the long-term coexistence of defector and cooperator genotypes also critically depends on the mode of dispersal and the dynamics of formation of social aggregation. If dispersal and colonisation happen by single cells, the survival of defectors is not possible since they are not able to build social groups alone. However, if dispersal happens through clusters of cells (e.g. by clusters of spores or by the detachment of larger fragments from the social group [47]) defector genotypes can be maintained. Social aggregation following dispersal of single cells can also lead to defector persistence [62].

Cheating and stronger forms of exploitation can undermine the stability of social groups and can even lead to the extinction of a social colony [63]. The presence of cheaters, presenting a 'cheater load' to the social group, is expected to lead to the evolution of strategies by which social groups can restrain or exclude cheaters. There are several theoretically possible mechanisms for cheater control, including intrinsic cheater inferiority or active policing strategies [65]. It is also possible that cheaters evolve ways to avoid the policing strategies that can lead to an evolutionary arms race between cheaters and cooperators.

Here I propose that the evolutionary transition from social prokaryotes to eukaryotic phagotrophs may have had transitory stages where protoeukaryotes played as cheaters in the social game of microbial biofilms. Cheaters later evolved into professional predators because of an ongoing evolutionary arms race between them and their cooperating host. I present a selective scenario for the origin of phagotrophy and discuss it from a game theoretical perspective. I also describe how this selective scenario can explain major cellular transformations during eukaryogenesis.

## Presentation of the hypothesis

### A social selective framework for the origin of phagotrophic exploitation

The ancestral feeding strategy of eukaryotes is based on the phagotrophic ingestion of other cells and intracellular digestion. I propose that such phagotrophic exploitation originated as a social cheater strategy in the interior of a social prokaryotic biofilm. Here I describe a selective scenario that can explain a gradual increase in the exploitation of a group of cell (the social host) by another one (the protoeukaryotes).

In the selective framework I propose, the relationship between cheater fitness and frequency is consistent with the fitness matrix of the chicken game (Fig. 1). With the increase of the magnitude of social exploitation during the origin of phagotrophy the relative magnitude of the fitness values remains unchanged (T>R>S>P) but the absolute values change (T increases, S decreases). When cheater abundance is low, cellular-level selection will favour cheater genotypes conferring higher levels of exploitation. Such 'local competition' is known to favour the spread of cheaters [66,67]. When cheaters are abundant, the density of the population decreases [68]. After reaching a critical cheater frequency, a colony will fail to produce dispersing cells and will go extinct. Colony-level selection or 'global competition' therefore restricts cheaters and promotes cooperation [67]. In this selective scenario, it is expected that cheater efficiency will continuously increase while genetic polymorphism is maintained. Parallel with increased exploitation the number of cheaters a social colony can support decreases. This way the overall cheater load of social groups remains constant. Cellular-level selection can thus drive an increase in exploitation while colony-level selection allows the long-term persistence of cheaters during several rounds of dispersal and social development.

I propose that in parallel with the evolution of cheating by protoeukaryotes the social host evolved active policing strategies [65]. By policing the host tried to discriminate, exclude or kill cheaters. As policing strategies evolved only those protoeukaryotes survived that were able to evolve mechanisms of avoidance. This situation could have led to an evolutionary arms race between the social host and the cheaters, similar to antagonistic coevolution between a host and its pathogens [69].

Below I describe how the two major selective factors, namely for increased exploitation and for the avoidance of policing strategies, could have driven cellular changes during eukaryogenesis.

### Cellular changes of eukaryogenesis driven by social conflict

Detailed, cell biologically sound evolutionary scenarios for many of the cellular changes during eukaryogenesis have been presented [4,5,13,21,70-72]. These scenarios are cell biologically realistic and are backed by bioinformatic and structural analyses of cellular components. I will here consider some critical major steps without much cell biological detail and place these in the social cheater scenario. It is therefore not the cell biological scenario that is novel here, but the attempt to define the social, ecological causation of these changes in more detail.

A plausible sequence of changes I propose is the following: social cheating, predation by extracellular digestion and diffuse nutrient uptake, cell wall loss, development of secretory and endocytic membranes, development of amoeboid motility allowing migration between colonies, development of phagotrophy to maximize feeding efficiency, uptake and enslavement of an alphaproteobacterium, switch from fermentation to respiration, origin of the nucleus triggered by mitochondrial symbiosis, development of cilia allowing planktonic predation, eukaryotic radiation. Although this sequence may seem arbitrary, the order of many of these steps is strongly constrained (e.g. phagotrophy could not have evolved in a cell with a rigid cell wall; cilia had to evolve after endomembranes etc., see [4,5,13,21,70-72] for further cell biological details).

Protoeukaryotic cheaters could have originated by reducing their contribution to a cooperatively produced fitness enhancing resource, e.g. by suppressing an altruistic autolysis program. Cheaters thus benefited from the altruism of social colony members without contributing equally. Later cheaters could have evolved mechanisms to actively trigger the autolysis of some host cells to further benefit from the excess nutrients released.

According to most models, the rigid prokaryotic cell wall had to be lost early during eukaryogenesis and it could have conferred resistance to antibiotics [20]. In the social cheater model, it can be explained as an adaptation to avoiding policing strategies targeting the cell wall. The social host could have evolved novel cell wall synthesis inhibitory antibiotics (and the necessary resistance traits) to exclude cheaters. Cheaters that survived cell wall synthesis inhibition (e.g. by internally rigidifying their plasmamembrane) avoided further cell wall policing. Cell wall loss was possible because the interior of biofilms provided osmotic, chemical and mechanical protection thereby buffering this radical cellular transformation. Subsequently protoeukaryotes could lyse host cells by secreting cell-wall digestive enzymes, without a risk of self-digestion. Cheaters thus evolved into cell wall-less predatory 'protoplasts' that externally digested other cells of the colony and took up diffusing nutrients. Secretion was not wasteful since biofilms limited diffusion and digestive enzymes could reach high concentrations.

After cell wall loss the plasmamembrane could be tubulated, vesiculated or protruded by the developing cytoskeleton. The cytoskeleton, evolving from filament systems present in prokaryotes [73-77], provided tensional integrity to the wall-less cells and a scaffold for vesicle trafficking. The first endomembranes were probably secretory tubules continuous with the plasma membrane that re-formed after each cell division [5]. The development of the first endomembranes allowed protoeukaryotes to secrete enzymes and to take up nutrients more efficiently. Novel policing strategies could also have contributed to driving endomembrane development. The host could have developed novel antibiotics to control protoeukaryotes. The signal peptidase, the signal peptide cleaving serine protease located on the extracellular side of the plasmamembrane, could have represented an easy antibiotic target (e.g. to arylomycins and beta-lactames [78,79]). Its inhibition could have provided a means for the host to interfere with protoeukaryote secretion. The signal peptidase of protoeukaryotes was directly exposed to the extracellular space and hiding it in intracellular tubules could have reduced its accessibility.

Lamellipodial motility, relying on actin-dependent membrane protrusion, adhesion and contraction, evolved to increase the efficiency of predation. For the first time in the history of life a flexible membrane and an underlying dynamic cytoskeleton could be used for protruding, adhering, sensing and signalling, and allowed protoeukaryotes to disperse actively inside the colony and also to invade other colonies by amoeboid motility on surfaces. Protoeukaryotes became generalist predators. With the evolution of active motility, protoeukaryotes relied less and less on passive dispersal (e.g. inside clusters of spores) and could actively forage their environment. As protoeukaryotes became independent from their social ancestor, their susceptibility to policing strategies also decreased. The selective forces became largely independent of the social context and further evolution was driven by the efficiency of predation.

Phagotrophy evolved as the most efficient system of predatory exploitation. It reduced the amount of digestive enzymes to be secreted and allowed the complete uptake of an engulfed cell's material [4]. I propose that besides providing more efficient food uptake, phagotrophy also had a decisive influence on protoeukaryote catabolism. In heterotrophs, generally there is a trade-off between the rate and the yield of ATP production (high rate but low yield versus low rate but high yield). One mole of glucose can for example be metabolised by respiration, yielding ~ 32 moles of ATP, but at a low rate. Glycolysis and fermentation yield only 2 moles of ATP per mole glucose, but at a higher rate [80]. If a cell uses a pathway with high yield and low rate it can produces more ATP per mole glucose and can consequently grow more from a given amount of resource. However, if such a cell is in resource competition with other cells that produce ATP at a higher rate but low overall yield, it will be in disadvantage [80]. The cells using the higher rate pathway (such as fermentation) will grow faster, even though they exploit the common resource inefficiently [81]. Generally if single-celled heterotrophs exploit external resources in a non-cooperative manner their growth-rate is maximal if they use reactions with the highest rate of ATP production even if at lower overall yield (e.g. fermentation of external sugars by *Lactobacilli *[80,82]). In contrast, if an internal resource is utilised (such as in animals or phagotrophs) using pathways with maximal ATP yield, even if at a lower rate, is expected to be favoured [80]. This could mean that with the evolution of internal resource use (phagotrophy) there was a strong selective pressure for pathways with higher yield of ATP production. Protoeukaryotic phagotrophs could have attained this by enslaving a respiring alphaproteobacterial prey. Phagotrophy and mitochondrial respiration were therefore synergistic in the evolution of efficient energy generation. Importantly, alphaproteobacterial symbiosis into a non-phagotrophic, fermenting osmotroph would not have had the same advantage than a phagotrophic fermenter could have had. This difference in the 'usefulness of respiration' between osmotrophs and phagotrophs gives therefore further theoretical support to phagotrophy-early scenarios.

Mitochondrial symbiosis could have initiated as an alphaproteobacterial phagotrophic prey circumvented digestion in the food vacuole. As predator pressure increased with the evolution of phagotrophy bacterial prey started to evolve pre- and post-ingestion defence mechanisms (and continues to do so ever since [83,84]). Examples of present proto/eukaryotic endosymbioses indicate that engulfed bacterial prey can escape digestion and establish a permanent presence in a phagotroph [85,86]. After the escape from the neutralised food vacuole alphaproteobacteria were enslaved by the insertion of a host-encoded ADP/ATP carrier. Using alphaproteobacterial respiration phagocytosed food could be converted into ATP with a high yield. The symbiont gradually evolved into an organelle via endosymbiotic gene transfer to the host genome [87] and the evolution of mitochondrial targeting mechanisms [88].

Mitochondrial symbiosis had a decisive impact on eukaryotic cell architecture. The distinct cytoplasmic, endoplasmic reticulum and mitochondrial versions of Hsp70 chaperons of alphaproteobacterial origin indicate that the final stages of ER evolution could have overlapped with the establishment of mitochondria as an organelle [4,89]. The spread of group II self-splicing introns from protomitochondria may also have triggered the evolution of telomeres and linear chromosomes [90]. Mitochondrial symbiosis probably also had a critical role in the evolution of nuclear compartmentalisation [72,91,92]. Nuclear compartmentalisation evolved when a tubular-vesicular membrane system surrounded chromatin and nuclear pores started to function as selective molecular sieves. The nuclear envelope evolved autogenously from pre-existing secretory endomembranes, while nuclear pores evolved by the modification of vesicle-curving complexes already present on endomembranes and functioning in vesicle budding [4,5,70].

Cilia evolved autogenously after the nucleus and mitochondria as sensory-motile organelles [71,93,94]. Cilium-based motility allowed eukaryotes to conquer pelagic habitats and to predate on planktonic prey. Long-term pelagic foraging was facilitated by other important adaptations including the origin of self-digestion (autophagy) that allowed survival under starvation conditions by digesting parts of the cytoplasm [95]. These adaptations to pelagic predation were probably the final major steps in eukaryogenesis. Having developed highly efficient ingestion, energy conversion, motility, and sensory systems eukaryotes rapidly diversified and quickly invaded most habitats where prokaryotic prey was present. The ubiquity of eukaryotic predation also prevented any further attempts to phagotrophy.

### The possible phylogenetic contexts of the emergence of protoeukaryotic cheaters

Given the widespread occurrence of social aggregation in prokaryotes [45,47,51,96-118] the hypothesis presented here in principle could fit into different phylogenetic settings. The aim of this paper is not to argue for any one of these in particular. Here I only briefly overview some of the more important phylogenetic frameworks that have been proposed and discuss how the cheater scenario could fit into them.

The mosaic nature of eukaryotic genomes consisting of genes of both eubacterial and archaebacterial affinity points to a fusion or symbiosis between members of these two groups [119-123]. Most parsimoniously, this can be explained as a merger of an archaebacterium (either stem or crown) and an alphaproteobacterium, the ancestor of mitochondria. A gammaproteobacterial [124] or myxobacterial [3] contribution to eukaryotes has also been proposed.

In the framework of the first scenario, social cheaters could have evolved from an archaebacterium in an archaebacterial biofilm. These cheaters then developed phagotrophy and internalised an alphaproteobacterium. Alternatively, cheaters may have developed in the stem lineage of the sister-groups archaebacteria and eukaryotes. According to the standard view of the tree of life archaebacteria or the stem archaebacteria/eukaryotes diverged from the last universal common ancestor independently from eubacteria (i.e. the universal tree is rooted between archaebacteria/eukaryotes and eubacteria).

In the 'neomuran' scenario [4,13] the stem lineage of archaebacteria/eukaryotes derives from a Gram-positive bacterium. This requires that the universal tree of life be rooted within Gram-negative bacteria for which strong arguments have recently been made [125]. In this scenario, wall-less cheaters were the ancestors of both archaebacteria (that re-evolved a rigid cell wall) and eukaryotes. Accordingly, cheaters could have evolved from Gram-positive, endospore forming bacteria. Phagotrophy could have evolved by the modification of the endospore formation pathway. There are striking similarities between the two processes. Both include the total engulfment of another cell by membrane invagination and fission. During both processes, the engulfing cell secretes hydrolytic enzymes to digest the septal cell wall (endospore formation) or the whole engulfed cell (phagocytosis). Interestingly, endospore formation can also be completed in the absence of the cell wall (i.e. by 'protoplasts') in *Bacillus *by adhesion and a ratchet mechanism [126].

In the myxobacterial scenario cheaters could have evolved from social myxobacteria [3,127]. As phagocytosis evolved an archaebacterium was internalised that evolved into the nucleus. An alphaproteobacterium was also phagocytosed subsequently and developed into mitochondria. Although many cell biological arguments can be made why an archaebacterium could not have evolved into a nucleus [21], it could still have provided archaebacterial genes.

## Testing the hypothesis

The selective scenario for increasing social exploitation, a crucial aspect of the hypothesis, can be tested using experimental model systems of social microbes (e.g. *Myxococcus*, *Bacillus*). The social cheater model proposes that an evolutionary arms race between protoeukaryotic cheaters and their host led to changing strategies and changing molecular mechanisms of cheating. It would be interesting to study experimentally how cheaters can be controlled by policing mechanisms and how new cheater genotypes can evolve under policing pressure. This could be done using serial competition experiments between cheater and social genotypes during several generations. In principle, there should be an escape from any policing strategy.

Most research on the evolution of social strategies is carried out using soil-dwelling microbes such as *Myxococcus *and *Dictyostelium*. It would be interesting to see whether social conflict can also originate in marine microbial biofilms since these were the first social systems to have evolved [128] and their study is probably also more relevant to the origin of eukaryotes. Such experiments could be carried out using both eubacterial (e.g. *Pseudomonas tunicata*) and archaebacterial marine biofilm forming species.

The social cheater model specifically proposes that cell wall-less protoeukaryotes as protoplasts had better chances of survival inside biofilms than they would have had as planktonic forms. This could be tested experimentally for example the following way: one could generate a temperature-sensitive mutant in a cell wall synthesis pathway gene that leads to cell wall loss at the restrictive temperature. This strain can be labelled with one resistance and can be mixed with a differently marked parental strain at the permissive temperature. The cells can then be grown either as a biofilm or a suspension culture. Following a shift to the restrictive temperature and further growth one could plate the cells and count the frequency of both types.

## Implications of the hypothesis

The importance of social conflict is widely appreciated in evolutionary biology. The social cheater hypothesis highlights the importance of social conflict in one of life's most dramatic evolutionary transitions, the origin of eukaryotic cells. The hypothesis also emphasizes how a social environment can buffer drastic cellular transformations that would be lethal for single-celled forms. The social framework can also provide a gradual cell evolutionary transition scenario from cheating to amoeboid and pelagic predation until the radiation of eukaryotes.

## Competing interests

The author declares that he has no competing interests.

## Reviewers' comments

### Reviewer's report 1

Eugene V Koonin National Center for Biotechnology Information, National Library of Medicine, National Institutes of Health, Bethesda, MD 20894, USA

The paper develops a scenario for the origin of eukaryotes under which protoeukaryotes started off as cheaters in prokaryotic biofilms, then shed the characteristic prokaryotic cell walls and so became phagocytes, then turned into "professional predators", and then engulfed the alpha-proteobacterial ancestor of the mitochondria; the mitochondrial endosymbiosis triggered the evolution of other major eukaryotic innovations, such as the nucleus.

This is not at all an unreasonable scenario, and if the principal message is that eukaryotes evolved in tightly knit microbial communities, such as biofilms, I tend to agree. Also, I accept the fact that all currently known eukaryotes have mitochondria or remnants thereof does not necessarily imply that the mitochondrial endosymbiosis was the event that triggered the emergence of all major eukaryotic innovations. It is, indeed, in principle, conceivable that phagocytosis antedated endosymbiosis. However, the specific succession of stages from a prokaryotic cheater to the protoeukaryote reads like a "just so story": everything is plausible but there is no specific evidence in support of any of the steps.

#### Author's response

I would say it is a constrained speculation rather then a "just so story". Any random order of events would hardly be as plausible evolutionarily as the one presented here. For example, it would be hard to argue that a cell with a rigid cell wall developed phagotrophy and endomembrane dynamics. The loss of the cell wall is a 'must' in any scenario. The order of the other events discussed in the transition scenario is substantiated by cell biology and molecular, structural and phylogenetic analyses, and I refer to the relevant literature, but don't repeat all these arguments (e.g. about the early origin of secretory endomembranes and the origin of the nuclear membrane from them, or the origin of cilia from a Golgi-like transport system, i.e. most likely after endomembranes). So many of the cell biological transitions are supported by evidence, of course only as good as it can be when we try to reconstruct past events from present patterns. I made this clearer in the revised text. The novel thing in this paper is not the cell biological scenario, but the attempt to find the social, ecological context of these changes.

Furthermore, the elaborate succession of steps is, exactly, what bothers me. If cheating is such an advantageous strategy and phagocytosis is so good for social prokaryotes, why are we not aware of any prokaryotes that have been locked during their evolution in one of these stages?

#### Author's response

Cheaters are abundantly present in social systems and can easily be generated in the laboratory. Phagotrophs, of course, not. What we don't know, how far cheaters can go in exploiting their host in nature. This could be partly addressed by long-term competition experiments as I suggested.

Generally, we do not know of any prokaryotic phagocytes which seems to be better compatible with the emergence of phagocytosis not before but after the critical event that was the start of eukaryogenesis...and acquisition of mitochondria looks like the best candidate for the role of such an event. So we go full circle: the hypothesis presented here is reasonable, even plausible, it is just that the evidence is not there. Or, is it the case that cheating prokaryotes capable of phagocytosis, actually, are there but are relatively rare and hard to cultivate? Perhaps, they could be discovered, e.g., in poorly studied archaeal biofilms? That would completely change the status of the present paper, and perhaps, even our understanding of the origin of eukaryotes in general. In the section on "Testing the hypothesis", it might be useful to be somewhat more explicit about this crucial validation of the proposed scenario.

#### Author's response

We don't know of any phagotrophic prokaryotes in nature, but we don't know of free-living mitochondrium-bearing prokaryotes either (locked at an intermediary stage). It means that exactly the same question about such a 'critical validation' can be posed about any scenario. However, this would be too cheap like this. I don't think there will be any phagotrophic prokaryotes discovered. Maybe the text gave the wrong impression that it was easy or fast to evolve phagotrophy. It was definitely not, and understanding it in detail may be one of the most difficult problems in cell evolution. What I tried to emphasize is that phagotrophy is the most crucial ecological novelty about eukaryotes and it is still extremely important and widespread today. Simply speaking, there was a huge prokaryotic biomass to be harvested by predators, and few would doubt that complete internalization is more efficient then external digestion and osmotrophy. There was a completely vacant niche to be filled, and it has been filled by the evolution of phagotrophic eukaryotes. Filled, *sensu strictu*, because there is hardly any place where there are prokaryotes, but no phagotrophic eukaryotes. So there is an ecological ceiling, and no prokaryote will again be able to break it to become phagotrophic. I also tried to devise a scenario that describes a gradual transition to phagotrophy through milder forms of exploitation.

About mitochondria as the best candidates: is there any evidence there that they came before endomembranes? I am not aware of any. This discussion somewhat reminds me of the 'contingency' versus 'convergence' debate of Gould and Conway Morris. According to the contingency scenario, the uptake of mitochondria was a unique and low-probability event that then triggered eukaryogenesis. In contrast, the convergence scenario would state that predators always appear when there is food to be ingested, i.e. eukaryogenesis was driven by well-known ecological principles, and only limited by the lack of mutations or external factors (e.g. high-enough oxygen concentration). I find the second perspective in this particular case more revealing.

Besides, mitochondria-early scenarios have very serious problems that have not yet been solved. How did symbiosis happen into a cell with a rigid cell wall? How did the origin of mitochondria trigger everything in eukaryogenesis? To date no realistic scenario exists that could explain why and how mitochondrial symbiosis led to the evolution of a secretory and endocytic membrane system, let alone phagotrophy. One would necessarily have to come back to scenarios such as the one here to explain how phagocytosis originated, because phagotrophy is what it is, from any perspective: the eating of other cells. So the selective pressure involved had to be the same: it was advantageous to eat. But then again: why only a cell with a mitochondrium could have evolved that capacity? I am not at all convinced about the necessity to have mitochondria in order to start to evolve a eukaryote. This view of looking at the problem is a reference to an extreme contingency: nothing could have happened until the uptake of the mitochondrium that then solved (almost) everything.

The section on "phylogenetic contexts", while including a disclaimer that this is not the central theme of the paper, reads too rudimentary and too "objective". I find it very strange that the scenario where the engulfing host is an archaeon (or a representative of a stem archaeal-eukaryotic lineage) is presented on exactly the same footing with two other scenarios that are, simply, unsupported. I am not trying to push any kind of agenda, I just believe that this is what the data point to.

#### Author's response

I am aware of the difficulties with this presentation. The point was to emphasize that the cheater scenario could in principle fit into any phylogenetic context. One can also see it as a historic overview of some important theories and phylogenetic propositions regarding eukaryote origins (I changed the text to make it clearer). However, regarding for example the scenario of Cavalier-Smith, I am not even convinced that it is 'simply unsupported'. To be able to make such a claim with confidence one would have to provide a detailed refutation of his polarizing arguments (evidence from evolutionary transition analyses) that point to a Gram-negative rooting of the tree of life [125]. Ignoring it or saying that it is not evidence is not a very brave strategy. If the rooting of Cavalier-Smith is correct, then Archaebacteria and Eukaryotes have to have derived from some kind of Eubacterium, most probably a Gram-positive lineage, and solid genomic evidence may be lacking because of the extremely derived status of the stem Archaebacterium/Eukaryote. The disclaimer therefore may also be interpreted in a way that I don't have a strong preference, but try to keep an open mind.

### Reviewer's report 2

Purificación López-García, Unité d'Ecologie, Systématique & Evolution, CNRS UMR 8079 Université Paris-Sud, bât. 360, 91405 Orsay Cedex, France

In this paper, G. Jékely proposes a hypothesis to explain the origin of phagotrophy, an essential property common to extant eukaryotic lines, in protoeukaryotic ancestors. The development of phagotrophy took place after the loss of the protoeukaryotic cell wall and led to important changes in cell architecture. The engulfment of one alphaproteobacterium that avoided digestion originated mitochondria and triggered eukaryogenesis. Phagotrophy originated in a social context, such as a microbial mat or biofilm where protoeukaryotes would behave as cheaters benefiting from the cooperative nature of the community without contributing to it, and was the consequence of two selective processes. The first involved the loss of the cell wall to escape antibiotics or other anti-cheater mechanisms targeting it. Once the cell wall was lost, phagocytosis would have evolved to maximize energy yield by incorporating preys and using them as an internal resource.

The proposal presented here is interesting. In fact, the idea that eukaryotes evolved in microbial mats, biofilms or other types of sedimentary social communities is implicit in some previous models (at least several symbiotic models).

#### Author's response

I cite the syntrophy-hypothesis and the hydrogen-hypothesis that indeed both imply some form of microbial community. One of the most important differences of the social cheater hypothesis is that is does not start with a mutually beneficial interaction and the gradual integration of two cells into one, but with exploitation of one cell by another one.

However, despite its interest, Jékely's idea is very difficult to test (see below) and does not provide information allowing discrimination between existing models for the origin of eukaryotes or an explanation for the origin of the eukaryotic nucleus, their key-defining character.

#### Author's response

The paper doesn't provide new information, but new arguments in favour of phagotrophy-early models and a new and detailed social-selective scenario for the origin of phagotrophy. It also helps to distinguish between models because it provides an ecologically sound, plausible historical narrative that tries to link ecological/selective causations with cell biological changes and, as a whole theory, can compete with previous models in its explanatory power (see also the section on historical narratives in my reply to Igor Zhulin). The autogenous origin of the nucleus (from pre-existing secretory membranes) can also be explained in this framework both cell biologically and regarding the selection pressures involved. I briefly discuss this and refer to some relevant literature, but a detailed model for the origin of the nucleus is beyond the scope of this paper.

A few specific comments follow.

1) I think there is a misuse of the term 'predation' as predation implies the ingestion (phagocytosis) of a prey. To me, 'digestion of neighboring cells', 'extracellular digestion and diffuse nutrient uptake' (referred to as predation in the manuscript) correspond to osmotrophy and, as such, are common to both prokaryotic and eukaryotic organisms. On the contrary, predation (implying phagotrophy) is a property exclusive of eukaryotes. The so-called 'predatory bacteria' such as Bdellovibrio spp. are not strictly predatory, but osmotrophic. They digest externally a larger prey into which they penetrate.

#### Author's response

It is a question about the definition of predation, and I would rather use the term in a broader sense, referring to the act of killing of other organisms (the prey) for food. Whether you eat it in pieces or as a whole, or whether you digest it externally or internally, are secondary in this regard (but not when efficiency is concerned).

2) Social cheaters are proposed to lose their cell walls as a way to escape to cheater-control strategies (e.g. antibiotics) developed by cooperative community members (referred as the host by Jékely, although this term may be also misleading). Wall-less cells would be buffered against osmotic crisis by the protecting biofilm environment. However, in nature many (if not most) microorganisms live in biofilms, mats or similar where social conflict occurs. If biofilms or mats host antibiotic producers that make antibiotics to fight against competitors or cheaters, antibiotics would have an increased concentration in the biofilm due to limited diffusion and microorganisms should cope with that. According to Jékely, cell-wall loss would be one advantageous adaptation. However, despite the fact that most microorganisms do form biofilms in nature, they do not lose their cell-walls. Why (frequent) cell-wall loss is not observed in natural biofilms or mats?

#### Author's response

I don't think we know how often cell wall-less mutants appear in biofilms and what is their short-term fate. What is worth mentioning here is that cells in a biofilm can undergo self-induced extensive genetic diversification by a recA-dependent mechanism (PNAS 2004 Nov 23;101(47):16630-5). This means that cell wall-less mutants probably appear more frequently in biofilms then in suspension. Whether this can happen to cheaters and enhance their success is not known. See also my response to Eugene Koonin about why cheaters will probably never again evolve into phagotrophs.

3) Jékely says that phagotrophy evolved as the most efficient system of predatory exploitation, and that this had to do with the yield of ATP production, which would be maximized if an internal resource were used. There would be 'a difference in the usefulness of respiration between osmotrophs and phagotrophs'. First, the latter is difficult to understand looking to nature, as O_2_-respiring bacterial osmotrophs are ecologically extremely successful. Second, I found the whole paragraph somewhat unclear, not knowing exactly if what was advantageous for the protoeukaryote was the phagocytosis of external food and its digestion as internal resource, the specific phagocytosis of mitochondrial ancestors and the acquisition of O_2_-respiration, or both. One possible reading is that Jékely suggests that this need to maximize ATP yield would have promoted the phagocytosis of mitochondrial O_2_-respiring ancestors within O_2_-respiring and/or fermentative protoeukaryotes (referred to as heterotrophs in the text). I can understand the advantage of acquiring O_2_-respiring mitochondria by a fermentative host and any other type of host using a less energy-yielding pathway (e.g. other types of respiration, methanogenesis would be one if O_2 _was not inhibitory for this pathway). However, I have more difficulties to understand why an O_2_-respiring single-celled organism (even if in community) needs to incorporate O_2_-respiring mitochondria. Organisms adapted to O_2_-respiration would respire rather than ferment given an appropriate O_2 _partial pressure exists. If O_2 _concentration is limiting for an O_2_-respiring host cell, it would be also limiting for an engulfed respiring alphaproteobacterium. The most likely explanation would be that mitochondria conferred O_2_-respiration to an organism that lacked it. This part of the hypothesis needs some clarification; perhaps, it would be worthwhile trying to describe better the nature of the protoeukaryotic ancestor, at least in metabolic terms.

#### Author's response

I re-phrased this paragraph to make it clearer. There are a few important things that I have to reiterate also here. It is not always true that "Organisms adapted to O_2_-respiration would respire rather than ferment given an appropriate O_2 _partial pressure exists". If yeast cells grow in direct competition on a fermentable resource (such as glucose) under aerobic conditions, it is not the respiring cells, but the fermenting ones that grow faster [81]. This is because fermentation has a higher ATP-generation rate then respiration, even if the yield per 1 mol glucose is lower. In other words fermentation is more efficient but also more wasteful then respiration. This is a 'tragedy of the commons' situation (see also [80]). Respiration is the winning strategy under other conditions, for example when non-fermentable carbon sources (e.g. lactate) are used, or when it pays off not to be wasteful, even if it is slower (e.g. utilizing resources in a spatially or temporally structured environment [81] or internal resources such as in animals and phagotrophs). Which pathway will provide a higher growth rate will therefore depend on many parameters and cells can also switch between the pathways, depending on the conditions. When osmotrophs became phagotrophs many of these parameters (e.g. the nature and availability of the carbon source) did not change, what changed is that the resource was used internally and not externally. And this change should have increased the selection for evolving or gaining respiration. That's what I meant when I wrote that respiration is more useful for a phagotroph then an osmotroph, all other parameters being equal. So it is easier to explain the origin of mitochondria in a phagotrophic and potentially still fermenting cell both mechanistically and in terms of the selection pressure driving symbiosis. Methanogenesis is also a possibility, but there is no evidence that it had anything to do with the last common ancestor of eukaryotes. Whereas we know that this ancestor had both aerobic mitochondrial respiration and phagotrophy.

4) Jékely also suggests several ways of testing part of this hypothesis. One consists to see whether an evolutionary arms race between cheaters and cooperative community members leads to changing strategies and molecular mechanisms of cheating. Another proposes to see whether cell wall-less mutants survive better in biofilms or in suspension. However, proving both (which, by the way, would appear likely) does not prove Jékely's hypothesis or even strongly favor it. It is difficult to test this model, though this does not invalidate it.

#### Author's response

These experiments would not prove that eukaryotes evolved the way I described, but they would greatly substantiate the selective scenario I proposed. Another very important test of a historical narrative is whether we can find a better, more plausible one, and more compatible with data. The few facts this scenario builds on are the following: (1) phagotrophic exploitation of prokaryotic biomass was present in the last common eukaryotic ancestor and its origin must have played an important role in eukaryogenesis. (2) Most prokaryotes live in social biofilms, and eukaryotes most likely evolved in a social setting. (3) Evolution is gradual, so phagotrophic exploitation should have evolved gradually through milder forms of exploitation. (4) A mild and widespread form of social exploitation in microbial biofilms is cheating. Having these four facts one only have to put them in order to have a meaningful historical narrative. That's what I have done here.

A third point mentioned by Jékely is to study social conflict in marine microbial biofilms 'since nutrient-rich terrestrial soils are not likely to have existed at the origin of eukaryotes'. I think this is rather gratuitous. We ignore exactly the environmental conditions at the time eukaryotes originated, and even when they appeared. They might or might not have evolved in marine environments. However, if we admit that eukaryotes appeared after prokaryotes, the latter had most likely colonized also continental substrates by the time eukaryotes evolved. Nutrient-rich environments for microorganisms have sense only at the microscale. I can very easily imagine many continental niches where biofilms and nutrient-rich substrates existed (including freshwater systems also), at least as nutrient-rich as the marine environment.

#### Author's response

You are right about this and I changed this sentence.

### Reviewer's report 3

Igor B. Zhulin, Joint Institute for Computational Sciences, The University of Tennessee – Oak Ridge National Laboratory, Oak Ridge, TN 37831-6173, USA

#### Preamble

I will start with the statement that I am certainly a wrong reviewer for this article. This was my first impression and, as we all know, it is always the right one. Still, I agreed to write a review, largely out of curiosity, and also because microbial biofilms are to a certain degree within my area of expertise. The second personal statement is that reading this essay made me understood why all decent universities keep molecular biologists and ecologist/evolutionary biologists in two separate departments: we speak different tongues... The final point for the preamble – I truly think that hypotheses should not be reviewed. They provide a reviewer with a wonderful opportunity to add after each "can", "may" and "if" sarcastic "... or cannot", "...or may not" and "... what if not"... Hypothesis is usually based on reasoning and serves a purpose of stimulating others in the field to test it (to find direct evidence for or against it). It is certainly not a reviewer's job to find "a dead body" and "a smoking gun".

#### Author's response

I don't think the only purpose of an evolutionary hypothesis is to stimulate others finding direct evidence for or against it. About many past events of evolution we will never have direct evidence (experimental, fossil, molecular or other). Should we then stop thinking about such events? Definitely not. As Ernst Mayr explains in his last books, What Makes Biology Unique? (page 32): "With the experiment unavailable for research in historical biology, a remarkable new heuristic method has been introduced, that of *historical narratives*. Just as in much of theory formation, the scientist starts with a conjecture and thoroughly tests it for its validity, so in evolutionary biology the scientist constructs a historical narrative, which is then tested for its explanatory value." In the end it is the explanatory value, the internal consistency, plausibility, and compatibility with data that counts. So in principle a reviewer can add after each "can", "may" and "if" sarcastic "... or cannot", "...or may not" and "... what if not"... and then see whether the resulting historical narrative has higher explanatory value.

#### Essence

As stated above, I am unfamiliar with the language and techniques of social ecology. To be completely honest, I dislike applying terms "cheating", "altruism", "self-sacrifice", "exploitation", "cooperation" to non-humans, especially to prokaryotes, simply because all these words require the presence of an intent. On the other hand, I am fully aware of the fact that these are legitimate definitions in ecology. Once again, I am against formal refereeing of hypotheses; therefore I will only focus on a couple of underlying postulates and propositions that are related to my area of expertise.

Table 1 is not needed. It is generally accepted now that almost all studied microbial species form biofilms. The list of species in this Table can be significantly expanded to include alpha-, beta-, and epsilon-proteobacteria and other clades (E. *coli*, *Salmonella*, *Burkholderia*, *Campylobacter*, *Listeria*, *Streptococcus*, *Nitrosomonas*, *Rhizobium*, *Treponema*, *Porphyromonas *and many-many others)

#### Author's response

I removed Table 1. The references to several examples of biofilm forming species among both archaebacteria and eubacteria are now in the main text.

The proposition that "the formation of biofilms is a social strategy that involves a costly contribution from cooperating individuals..." (page 5, last paragraph) does not have experimental support. Quite a few genes are both up- and down-regulated in both planktonic cultures and biofilms: their expression profiles are very different (for example, see BMC Genomics 2006, 7: 162), but I have never seen data suggesting that it is more costly for a cell to live in a biofilm. Yes, cells in biofilms excrete more "stuff" into the matrix, but, for instance, in contrast to cells in biofilms, planktonic cells produce dozens of proteins in quite some numbers to build their flagella and use a significant percentage of their total energy to power the flagellar motor. Nobody ever tried to make any decent calculations (it will be quite some work) to determine whether it costs more to be free or to be stuck in the biofilm!

#### Author's response

The point is not to compare the costs of forming biofilms versus growing in suspension, but contributing to the formation and dispersal of biofilms versus not contributing to it, yet living in it and enjoying its benefits. So it is less costly to sit in a biofilm, without secreting the costly matrix, then sitting in it and secreting the matrix. In this sense the social strategy (to contribute) is costly. The sparing of this cost can lead to the spread of defectors and cheaters because if they don't contribute to this 'public good' they can grow faster. There is experimental demonstration of the invasion of a biofilm by cheaters that don't contribute to an extracellular cellulosic polymer [49]. It is also to be noted, though, that extracellular matrix production can also be interpreted as a competitive and not cooperative strategy [50]. Even if it will turn out to be the case in some biofilms, there are also other social aspects of biofilm formation and dispersal, as I explained in the text.

This hypothesis would be very difficult to test. Suggested experiment (survival of protoplasts in the biofilm matrix) will be quite difficult (if at all possible) to implement (as a relatively recent experimental microbiologist I cannot see how it can be done technically), and even it is successful, the experiment will fall short of being considered as evidence.

#### Author's response

Unless one can re-evolve a eukaryote, none of the microbial experiments I proposed can be considered as decisive evidence. They would only substantiate some of my assumptions. Regarding some of the experimental tests, I am more optimistic, though, and now included a more detailed suggestion for the protoplast experiment.

I would like to finish on a more positive note. This was a very entertaining reading and this is a good hypothesis: I certainly cannot disprove it!

#### Author's response

This is good to hear. I don't think any (not totally unreasonable) historical narrative can ever be disproved in the strict sense. The question is if one can suggest a better one.

## References

[B1] Maynard Smith J, Szathmáry E (1995). The Major Transitions in Evolution.

[B2] Martin W, Muller M (1998). The hydrogen hypothesis for the first eukaryote. Nature.

[B3] Moreira D, Lopez-Garcia P (1998). Symbiosis between methanogenic archaea and delta-proteobacteria as the origin of eukaryotes: the syntrophic hypothesis. J Mol Evol.

[B4] Cavalier-Smith T (2002). The phagotrophic origin of eukaryotes and phylogenetic classification of Protozoa. Int J Syst Evol Microbiol.

[B5] Jékely G (2003). Small GTPases and the evolution of the eukaryotic cell. Bioessays.

[B6] Embley TM, Martin W (2006). Eukaryotic evolution, changes and challenges. Nature.

[B7] Hirt RP, Horner DS (2004). Organelles, genomes and eukaryote phylogeny: An Evolutionary Synthesis in the Age of Genomics.

[B8] Richards TA, Cavalier-Smith T (2005). Myosin domain evolution and the primary divergence of eukaryotes. Nature.

[B9] Jékely G, (Ed) (2006). Origins and evolution of eukaryotic endomembranes and cytoskeleton.

[B10] Vellai T, Takacs K, Vida G (1998). A new aspect to the origin and evolution of eukaryotes. J Mol Evol.

[B11] Lopez-Garcia P, Moreira D (1999). Metabolic symbiosis at the origin of eukaryotes. Trends Biochem Sci.

[B12] Emelyanov VV (2003). Mitochondrial connection to the origin of the eukaryotic cell. Eur J Biochem.

[B13] Cavalier-Smith T (1987). The origin of eukaryotic and archaebacterial cells. Ann N Y Acad Sci.

[B14] Clark CG, Roger AJ (1995). Direct evidence for secondary loss of mitochondria in Entamoeba histolytica. Proc Natl Acad Sci USA.

[B15] Roger AJ, Clark CG, Doolittle WF (1996). A possible mitochondrial gene in the early-branching amitochondriate protist Trichomonas vaginalis. Proc Natl Acad Sci USA.

[B16] Germot A, Philippe H, Le Guyader H (1996). Presence of a mitochondrial-type 70-kDa heat shock protein in Trichomonas vaginalis suggests a very early mitochondrial endosymbiosis in eukaryotes. Proc Natl Acad Sci USA.

[B17] Germot A, Philippe H, Le Guyader H (1997). Evidence for loss of mitochondria in Microsporidia from a mitochondrial-type HSP70 in Nosema locustae. Mol Biochem Parasitol.

[B18] Roger AJ, Svard SG, Tovar J, Clark CG, Smith MW, Gillin FD, Sogin ML (1998). A mitochondrial-like chaperonin 60 gene in Giardia lamblia: evidence that diplomonads once harbored an endosymbiont related to the progenitor of mitochondria. Proc Natl Acad Sci USA.

[B19] Gray MW, Burger G, Lang BF (1999). Mitochondrial evolution. Science.

[B20] Cavalier-Smith T (2002). The neomuran origin of archaebacteria, the negibacterial root of the universal tree and bacterial megaclassification. Int J Syst Evol Microbiol.

[B21] Jékely G, Jékely G (2006). Origin of eukaryotic endomembranes – a critical evaluation of different model scenarios. Origins and evolution of eukaryotic endomembranes and cytoskeleton.

[B22] Simpson AG, Roger AJ (2004). The real 'kingdoms' of eukaryotes. Curr Biol.

[B23] Cohen CJ, Bacon R, Clarke M, Joiner K, Mellman I (1994). Dictyostelium discoideum mutants with conditional defects in phagocytosis. J Cell Biol.

[B24] Boenigk J, Arndt H (2002). Bacterivory by heterotrophic flagellates: community structure and feeding strategies. Antonie Van Leeuwenhoek.

[B25] Steenkamp ET, Wright J, Baldauf SL (2006). The protistan origins of animals and fungi. Mol Biol Evol.

[B26] James TY, Kauff F, Schoch CL, Matheny PB, Hofstetter V, Cox CJ, Celio G, Gueidan C, Fraker E, Miadlikowska J (2006). Reconstructing the early evolution of Fungi using a six-gene phylogeny. Nature.

[B27] Powell MJ (1984). Fine structure of the unwalled thallus of Rozella polyphagi in its host Polyphagus euglenae. Mycologia.

[B28] Stechmann A, Cavalier-Smith T (2002). Rooting the eukaryote tree by using a derived gene fusion. Science.

[B29] Cavalier-Smith T, Chao EE (2003). Phylogeny of choanozoa, apusozoa, and other protozoa and early eukaryote megaevolution. J Mol Evol.

[B30] Nikolaev SI, Berney C, Fahrni JF, Bolivar I, Polet S, Mylnikov AP, Aleshin VV, Petrov NB, Pawlowski J (2004). The twilight of Heliozoa and rise of Rhizaria, an emerging supergroup of amoeboid eukaryotes. Proc Natl Acad Sci USA.

[B31] Burki F, Pawlowski J (2006). Monophyly of Rhizaria and multigene phylogeny of unicellular bikonts. Mol Biol Evol.

[B32] Simpson AG (2003). Cytoskeletal organization, phylogenetic affinities and systematics in the contentious taxon Excavata (Eukaryota). Int J Syst Evol Microbiol.

[B33] Cavalier-Smith T, Chao EE (2006). Phylogeny and megasystematics of phagotrophic heterokonts (kingdom Chromista). J Mol Evol.

[B34] Brugerolle G (1975). Ultrastructure of the genus Enteromonas da Fonseca (Zoomastigophorea) and revision of the order of Diplomonadida Wenyon. J Protozool.

[B35] Sogayar MI, Gregorio EA (1989). Uptake of bacteria by trophozoites of Giardia duodenalis (Say). Ann Trop Med Parasitol.

[B36] Pereira-Neves A, Benchimol M (2006). Phagocytosis by Trichomonas vaginalis – New Insights. Biol Cell.

[B37] Cermakian N, Ikeda TM, Miramontes P, Lang BF, Gray MW, Cedergren R (1997). On the evolution of the single-subunit RNA polymerases. J Mol Evol.

[B38] Brinkmann H, Philippe H, Jékely G (2006). The Diversity of Eukaryotes and the Root of the Eukaryotic Tree. Origins and Evolution of Eukaryotic Endomembranes and Cytoskeleton.

[B39] Flavin M, Nerad TA (1993). Reclinomonas americana N. G., N. Sp., a new freshwater heterotrophic flagellate. J Eukaryot Microbiol.

[B40] Stoodley P, Sauer K, Davies DG, Costerton JW (2002). Biofilms as complex differentiated communities. Annu Rev Microbiol.

[B41] Webb JS, Givskov M, Kjelleberg S (2003). Bacterial biofilms: prokaryotic adventures in multicellularity. Curr Opin Microbiol.

[B42] Hall-Stoodley L, Costerton JW, Stoodley P (2004). Bacterial biofilms: from the natural environment to infectious diseases. Nat Rev Microbiol.

[B43] Branda SS, Vik S, Friedman L, Kolter R (2005). Biofilms: the matrix revisited. Trends Microbiol.

[B44] Stanley NR, Britton RA, Grossman AD, Lazazzera BA (2003). Identification of catabolite repression as a physiological regulator of biofilm formation by Bacillus subtilis by use of DNA microarrays. J Bacteriol.

[B45] Pysz MA, Conners SB, Montero CI, Shockley KR, Johnson MR, Ward DE, Kelly RM (2004). Transcriptional analysis of biofilm formation processes in the anaerobic, hyperthermophilic bacterium Thermotoga maritima. Appl Environ Microbiol.

[B46] O'Connor KA, Zusman DR (1991). Development in Myxococcus xanthus involves differentiation into two cell types, peripheral rods and spores. J Bacteriol.

[B47] Mai-Prochnow A, Evans F, Dalisay-Saludes D, Stelzer S, Egan S, James S, Webb JS, Kjelleberg S (2004). Biofilm development and cell death in the marine bacterium Pseudoalteromonas tunicata. Appl Environ Microbiol.

[B48] Lombardia E, Rovetto AJ, Arabolaza AL, Grau RR (2006). A LuxS-dependent cell-to-cell language regulates social behavior and development in Bacillus subtilis. J Bacteriol.

[B49] Rainey PB, Rainey K (2003). Evolution of cooperation and conflict in experimental bacterial populations. Nature.

[B50] Xavier JB, Foster KR (2007). Cooperation and conflict in microbial biofilms. Proc Natl Acad Sci USA.

[B51] Lapaglia C, Hartzell PL (1997). Stress-Induced Production of Biofilm in the Hyperthermophile Archaeoglobus fulgidus. Appl Environ Microbiol.

[B52] Costerton JW, Stewart PS, Greenberg EP (1999). Bacterial biofilms: a common cause of persistent infections. Science.

[B53] Matz C, McDougald D, Moreno AM, Yung PY, Yildiz FH, Kjelleberg S (2005). Biofilm formation and phenotypic variation enhance predation-driven persistence of Vibrio cholerae. Proc Natl Acad Sci USA.

[B54] Brockhurst MA, Hochberg ME, Bell T, Buckling A (2006). Character displacement promotes cooperation in bacterial biofilms. Curr Biol.

[B55] Mai-Prochnow A, Webb JS, Ferrari BC, Kjelleberg S (2006). Ecological advantages of autolysis during the development and dispersal of Pseudoalteromonas tunicata biofilms. Appl Environ Microbiol.

[B56] Gonzalez-Pastor JE, Hobbs EC, Losick R (2003). Cannibalism by sporulating bacteria. Science.

[B57] Strassmann JE, Zhu Y, Queller DC (2000). Altruism and social cheating in the social amoeba Dictyostelium discoideum. Nature.

[B58] Velicer GJ, Kroos L, Lenski RE (2000). Developmental cheating in the social bacterium Myxococcus xanthus. Nature.

[B59] Vulic M, Kolter R (2001). Evolutionary cheating in Escherichia coli stationary phase cultures. Genetics.

[B60] Turner PE, Chao L (1999). Prisoner's dilemma in an RNA virus. Nature.

[B61] Velicer GJ (2003). Social strife in the microbial world. Trends Microbiol.

[B62] Matapurkar AK, Watve MG (1997). Altruist cheater dynamics in Dictyostelium: aggregated distribution gives stable oscillations. The American Naturalist.

[B63] Fiegna F, Velicer GJ (2003). Competitive fates of bacterial social parasites: persistence and self-induced extinction of Myxococcus xanthus cheaters. Proc Biol Sci.

[B64] Axelrod R, Hamilton WD (1981). The evolution of cooperation. Science.

[B65] Travisano M, Velicer GJ (2004). Strategies of microbial cheater control. Trends Microbiol.

[B66] West SA, Buckling A (2003). Cooperation, virulence and siderophore production in bacterial parasites. Proc Biol Sci.

[B67] Griffin AS, West SA, Buckling A (2004). Cooperation and competition in pathogenic bacteria. Nature.

[B68] Harrison F, Buckling A (2005). Hypermutability impedes cooperation in pathogenic bacteria. Curr Biol.

[B69] Buckling A, Rainey PB (2002). Antagonistic coevolution between a bacterium and a bacteriophage. Proc Biol Sci.

[B70] Devos D, Dokudovskaya S, Alber F, Williams R, Chait BT, Sali A, Rout MP (2004). Components of coated vesicles and nuclear pore complexes share a common molecular architecture. PLoS Biol.

[B71] Jékely G, Arendt D (2006). Evolution of intraflagellar transport from coated vesicles and autogenous origin of the eukaryotic cilium. Bioessays.

[B72] Martin W, Koonin EV (2006). Introns and the origin of nucleus-cytosol compartmentalization. Nature.

[B73] Lowe J, Amos LA (1998). Crystal structure of the bacterial cell-division protein FtsZ. Nature.

[B74] Lowe J, Amos LA (1999). Tubulin-like protofilaments in Ca2+-induced FtsZ sheets. Embo J.

[B75] Ben-Yehuda S, Losick R (2002). Asymmetric cell division in B. subtilis involves a spiral-like intermediate of the cytokinetic protein FtsZ. Cell.

[B76] van den Ent F, Amos L, Lowe J (2001). Bacterial ancestry of actin and tubulin. Curr Opin Microbiol.

[B77] Moller-Jensen J, Jensen RB, Lowe J, Gerdes K (2002). Prokaryotic DNA segregation by an actin-like filament. Embo J.

[B78] Paetzel M, Goodall JJ, Kania M, Dalbey RE, Page MG (2004). Crystallographic and biophysical analysis of a bacterial signal peptidase in complex with a lipopeptide-based inhibitor. J Biol Chem.

[B79] Paetzel M, Dalbey RE, Strynadka NC (1998). Crystal structure of a bacterial signal peptidase in complex with a beta-lactam inhibitor. Nature.

[B80] Pfeiffer T, Schuster S, Bonhoeffer S (2001). Cooperation and competition in the evolution of ATP-producing pathways. Science.

[B81] MacLean RC, Gudelj I (2006). Resource competition and social conflict in experimental populations of yeast. Nature.

[B82] Poolman B (1993). Energy transduction in lactic acid bacteria. FEMS Microbiol Rev.

[B83] Matz C, Kjelleberg S (2005). Off the hook – how bacteria survive protozoan grazing. Trends Microbiol.

[B84] Pernthaler J (2005). Predation on prokaryotes in the water column and its ecological implications. Nat Rev Microbiol.

[B85] Fenchel T, Bernard C (1993). Endosymbiotic purple non-sulphur bacteria in an anaerobic ciliated protozoon. FEMS Microbiol Lett.

[B86] Fenchel T, Bernard C (1993). A purple protist. Nature.

[B87] Lang BF, Gray MW, Burger G (1999). Mitochondrial genome evolution and the origin of eukaryotes. Annu Rev Genet.

[B88] Cavalier-Smith T (2006). Origin of mitochondria by intracellular enslavement of a photosynthetic purple bacterium. Proc Biol Sci.

[B89] Gupta RS, Aitken K, Falah M, Singh B (1994). Cloning of Giardia lamblia heat shock protein HSP70 homologs: implications regarding origin of eukaryotic cells and of endoplasmic reticulum. Proc Natl Acad Sci USA.

[B90] Koonin EV (2006). The origin of introns and their role in eukaryogenesis: a compromise solution to the introns-early versus introns-late debate?. Biol Direct.

[B91] Mans BJ, Anantharaman V, Aravind L, Koonin EV (2004). Comparative genomics, evolution and origins of the nuclear envelope and nuclear pore complex. Cell Cycle.

[B92] Jékely G (2005). Glimpsing over the event horizon: evolution of nuclear pores and envelope. Cell Cycle.

[B93] Mitchell DR (2004). Speculations on the evolution of 9+2 organelles and the role of central pair microtubules. Biol Cell.

[B94] Mitchell DR, Jékely G (2006). The evolution of eukaryotic cilia and flagella as motile and sensory organelles. Origins and Evolution of Eukaryotic Endomembranes and Cytoskeleton.

[B95] Hughes T, Rusten TE, Jékely G (2006). Origin and evolution of self-consumption: autophagy. Origins and Evolution of Eukaryotic Endomembranes and Cytoskeleton.

[B96] Alvarez B, Secades P, Prieto M, McBride MJ, Guijarro JA (2006). A mutation in Flavobacterium psychrophilum tlpB inhibits gliding motility and induces biofilm formation. Appl Environ Microbiol.

[B97] Battin TJ, Wille A, Sattler B, Psenner R (2001). Phylogenetic and functional heterogeneity of sediment biofilms along environmental gradients in a glacial stream. Appl Environ Microbiol.

[B98] Branda SS, Chu F, Kearns DB, Losick R, Kolter R (2006). A major protein component of the Bacillus subtilis biofilm matrix. Mol Microbiol.

[B99] Brummer IH, Felske AD, Wagner-Dobler I (2004). Diversity and seasonal changes of uncultured Planctomycetales in river biofilms. Appl Environ Microbiol.

[B100] Dana JR, Shimkets LJ (1993). Regulation of cohesion-dependent cell interactions in Myxococcus xanthus. J Bacteriol.

[B101] Hamon MA, Lazazzera BA (2001). The sporulation transcription factor Spo0A is required for biofilm development in Bacillus subtilis. Mol Microbiol.

[B102] Henneberger R, Moissl C, Amann T, Rudolph C, Huber R (2006). New insights into the lifestyle of the cold-loving SM1 euryarchaeon: natural growth as a monospecies biofilm in the subsurface. Appl Environ Microbiol.

[B103] Edwards KJ, Bond PL, Gihring TM, Banfield JF (2000). An archaeal iron-oxidizing extreme acidophile important in acid mine drainage. Science.

[B104] Bond PL, Smriga SP, Banfield JF (2000). Phylogeny of microorganisms populating a thick, subaerial, predominantly lithotrophic biofilm at an extreme acid mine drainage site. Appl Environ Microbiol.

[B105] Kim YM, Kim JH (2004). Formation and dispersion of mycelial pellets of Streptomyces coelicolor A3(2). J Microbiol.

[B106] Koch M, Rudolph C, Moissl C, Huber R (2006). A cold-loving crenarchaeon is a substantial part of a novel microbial community in cold sulphidic marsh water. FEMS Microbiol Ecol.

[B107] Nather DJ, Rachel R, Wanner G, Wirth R (2006). Flagella of Pyrococcus furiosus: multifunctional organelles, made for swimming, adhesion to various surfaces, and cell-cell contacts. J Bacteriol.

[B108] Tyson GW, Chapman J, Hugenholtz P, Allen EE, Ram RJ, Richardson PM, Solovyev VV, Rubin EM, Rokhsar DS, Banfield JF (2004). Community structure and metabolism through reconstruction of microbial genomes from the environment. Nature.

[B109] Ram RJ, Verberkmoes NC, Thelen MP, Tyson GW, Baker BJ, Blake RC, Shah M, Hettich RL, Banfield JF (2005). Community proteomics of a natural microbial biofilm. Science.

[B110] Rinker KD, Kelly RM (1996). Growth Physiology of the Hyperthermophilic Archaeon Thermococcus litoralis: Development of a Sulfur-Free Defined Medium, Characterization of an Exopolysaccharide, and Evidence of Biofilm Formation. Appl Environ Microbiol.

[B111] Schrenk MO, Kelley DS, Bolton SA, Baross JA (2004). Low archaeal diversity linked to subseafloor geochemical processes at the Lost City Hydrothermal Field, Mid-Atlantic Ridge. Environ Microbiol.

[B112] van der Meer MT, Schouten S, de Leeuw JW, Ward DM (2000). Autotrophy of green non-sulphur bacteria in hot spring microbial mats: biological explanations for isotopically heavy organic carbon in the geological record. Environ Microbiol.

[B113] Weidler GW, Dornmayr-Pfaffenhuemer M, Gerbl FW, Heinen W, Stan-Lotter H (2006). Communities of Archaea and Bacteria in a subsurface radioactive thermal spring in the Austrian Central Alps and evidence for ammonia oxidizing Crenarchaeota. Appl Environ Microbiol.

[B114] Webb JS, Thompson LS, James S, Charlton T, Tolker-Nielsen T, Koch B, Givskov M, Kjelleberg S (2003). Cell death in Pseudomonas aeruginosa biofilm development. J Bacteriol.

[B115] Watnick PI, Kolter R (1999). Steps in the development of a Vibrio cholerae El Tor biofilm. Mol Microbiol.

[B116] Webster NS, Negri AP (2006). Site-specific variation in Antarctic marine biofilms established on artificial surfaces. Environ Microbiol.

[B117] Zhang T, Fang HH (2001). Phylogenetic diversity of a SRB-rich marine biofilm. Appl Microbiol Biotechnol.

[B118] Zhang T, Fang HH, Ko BC (2003). Methanogen population in a marine biofilm corrosive to mild steel. Appl Microbiol Biotechnol.

[B119] Golding GB, Gupta RS (1995). Protein-based phylogenies support a chimeric origin for the eukaryotic genome. Mol Biol Evol.

[B120] Brown JR, Doolittle WF (1997). Archaea and the prokaryote-to-eukaryote transition. Microbiol Mol Biol Rev.

[B121] Gupta RS (1998). Protein phylogenies and signature sequences: A reappraisal of evolutionary relationships among archaebacteria, eubacteria, and eukaryotes. Microbiol Mol Biol Rev.

[B122] Ribeiro S, Golding GB (1998). The mosaic nature of the eukaryotic nucleus. Mol Biol Evol.

[B123] Rivera MC, Lake JA (2004). The ring of life provides evidence for a genome fusion origin of eukaryotes. Nature.

[B124] Horiike T, Hamada K, Miyata D, Shinozawa T (2004). The origin of eukaryotes is suggested as the symbiosis of pyrococcus into gamma-proteobacteria by phylogenetic tree based on gene content. J Mol Evol.

[B125] Cavalier-Smith T (2006). Rooting the tree of life by transition analyses. Biol Direct.

[B126] Broder DH, Pogliano K (2006). Forespore engulfment mediated by a ratchet-like mechanism. Cell.

[B127] Lopez-Garcia P, Moreira D (2006). Selective forces for the origin of the eukaryotic nucleus. Bioessays.

[B128] Schopf JW (2006). Fossil evidence of Archaean life. Philos Trans R Soc Lond B Biol Sci.

